# Building a Medical Education Outcomes Center: Development Study

**DOI:** 10.2196/14651

**Published:** 2019-10-31

**Authors:** Mark E Rosenberg, Jacqueline L Gauer, Barbara Smith, Austin Calhoun, Andrew P J Olson, Emily Melcher

**Affiliations:** 1 Office of Medical Education University of Minnesota Medical School Minneapolis, MN United States; 2 Office of Health Sciences Technology University of Minnesota Minneapolis, MN United States

**Keywords:** outcome measures, data analysis, physicians, medical students, database management systems, data linkage

## Abstract

**Background:**

Medical education outcomes and clinical data exist in multiple unconnected databases, resulting in 3 problems: (1) it is difficult to connect learner outcomes with patient outcomes, (2) learners cannot be easily tracked over time through the education-training-practice continuum, and (3) no standard methodology ensures quality and privacy of the data.

**Objective:**

The purpose of this study was to develop a Medical Education Outcomes Center (MEOC) to integrate education data and to build a framework to standardize the intake and processing of requests for using these data.

**Methods:**

An inventory of over 100 data sources owned or utilized by the medical school was conducted, and nearly 2 dozen of these data sources have been vetted and integrated into the MEOC. In addition, the American Medical Association (AMA) Physician Masterfile data of the University of Minnesota Medical School (UMMS) graduates were linked to the data from the National Provider Identifier (NPI) registry to develop a mechanism to connect alumni practice data to education data.

**Results:**

Over 160 data requests have been fulfilled, culminating in a range of outcomes analyses, including support of accreditation efforts. The MEOC received data on 13,092 UMMS graduates in the AMA Physician Masterfile and could link 10,443 with NPI numbers and began to explore their practice demographics. The technical and operational work to expand the MEOC continues. Next steps are to link the educational data to the clinical practice data through NPI numbers to assess the effectiveness of our medical education programs by the clinical outcomes of our graduates.

**Conclusions:**

The MEOC provides a replicable framework to allow other schools to more effectively operate their programs and drive innovation.

## Introduction

### Background

In this era of *big data* and advanced data analytics, medical education must more effectively utilize data to enhance pedagogy, advance scholarship, and link educational outcomes to clinical outcomes [1-8]. This involves the integration of noneducational data, such as clinical practice data, into the evaluation of medical education programs [3-8]. An essential goal of medical education evaluation is to ultimately determine the quality of our medical education programs by the quality of care delivered by our graduates and trainees. Data should also be used to develop and guide teaching and learning, facilitate curricular development, and optimize educational experiences to develop future physicians who are diverse, meet workforce needs, and can positively impact health outcomes.

The vast amount of data generated and collected by medical schools has the potential to transform innovation in medical education. However, these valuable data often languish in siloed databases, making them inaccessible to those who need them the most. In addition, many of these databases lack standardized methodology and processes to guarantee that data are of high quality and that data security is maintained [1,2,8,9]. A system for tracking learners as they progress along the medical education continuum and into practice remains a challenge, as does determining which clinical practice outcomes are most sensitive to educational intervention effectiveness.

### Objectives

In response to these challenges, we developed the Medical Education Outcomes Center (MEOC) to connect educators, researchers, and other stakeholders to education researchers, datasets, data experts, and innovative analyses. We sought to collect, integrate, and manage data to enhance medical education programs, including strategic decision making and quality improvement, and to advance medical education scholarship. As part of these efforts, we developed a data request framework to efficiently and ethically receive and fulfill requests for medical school data. Finally, we identified a need to connect education data to practice data of our graduates. The purpose of this paper is to describe how we created MEOC, so that other institutions might replicate our process to meet the challenges of data integration, access, and utilization.

## Methods

### Resourcing and Personnel

As we considered tracking outcomes as a fundamental operational issue of a medical school, we resourced MEOC through medical school operational funds. Therefore, the MEOC team’s structure is lean, and many members wear several hats. During the developmental phase of the project, the project team comprised a project sponsor, project manager, technical owner, business owner, website developer, and 1 analyst each from the Office of Medical Education and the Office of Health Sciences Technology. Aside from the analyst, none of the project team members were dedicated more than half-time to the MEOC project. This lean team structure provides a model for other institutions that may also wish to develop an outcomes center on a limited budget. Now that the MEOC has been launched, the team is growing and is exploring additional avenues for funding, including extramural grants.

The developmental phase of the MEOC project, including convening the initial team, inventorying of data sources, devising the request and data governance framework, and building the website, lasted approximately 1 year. Much of the work creating the request framework could be short-circuited by other institutions by following the model already devised by MEOC.

### The Medical Education Outcomes Center’s Data Framework

One of the goals of the MEOC is to provide a centralized resource for all data and data-related services within the medical school. As the requesters’ data-related needs can be varied, specific, and occasionally unpredictable, the data model followed by the MEOC was initially developed to be flexible and responsive to the unique needs of every requester. Therefore, instead of collecting multiple data elements into a single, highly structured database as in a traditional data warehouse model, under the initial MEOC model, all data continue to reside in their original data sources. By using standardized, reusable code and logic when feasible, these data are accessed and combined on an ad hoc basis by the MEOC data analysts. MEOC provides structure to the data model by inventorying, documenting, vetting, and validating the data sources to integrate them into the MEOC data framework. Furthermore, every fulfilled data request is carefully documented, which allows for increasingly efficient replication of frequently used data combinations. This model allows for bespoke combinations of data sources and elements as well as on-the-fly integration of new data sources as necessary. In addition, this initial approach allowed us to move forward with the MEOC development efforts relatively quickly and allowed us to understand and assess how frequently and in what ways the various data sources and data elements were routinely utilized. Through this approach, we are now able to assess and understand the needs, requirements, and resources to potentially develop a data warehouse.

### Data Elements and Sources

To lay a strong foundation for the data framework described above, the MEOC staff conducted a comprehensive inventory of over 100 data sources owned or utilized by the medical school, and nearly 2 dozen of these data sources have been vetted and integrated to date (Table 1). Disparate data sources were integrated with one another using common identifiers, data feeds, structured query language, and database views. Data sources were also connected using Tableau Server (Tableau Software, Inc, Seattle, WA) [10], a software tool that allows us to easily connect to and link together a variety of different data formats and relational database management systems.

We performed quality assurance of the data by applying data normalization and cleaning techniques to minimize or eliminate redundant data; to standardize data to account for the changes in how values were captured within certain data fields over the years; to account for inconsistencies in data owing to migration issues in which data were previously imported incorrectly; and to convert numeric, date, or character data types where necessary. Part of this process involved arranging data into logical groupings and promoting standardization across data sources. Potential limitations, constraints, anomalies, or notable exceptions about the data were identified, and the best practices for the use of the data were suggested. We captured this information as *meta-references* for each data source within an administrative metadata section of our MEOC website and also created data dictionaries and other supporting metadata documentation for each data source. We worked with subject matter experts to translate key definitions into data terms to establish and document accurate, shared data definitions.

**Table 1:** Examples of data integrated into the Medical Education Outcomes Center.

Data types	Data sources (examples)
Prematriculation data	American Medical College Application Service Integrated Postsecondary Education Data System Medical Scientist Training Program—includes admissions and assessments data MedAdmissions—University of Minnesota Medical School admissions data, including supplemental applications, interview, and other selection information
Undergraduate medical education	BlackBag—learning management system containing assignment, assessment, and curriculum data CoursEval—course and instructor evaluations, year-end evaluations, self-assessment, peer assessment, midcourse feedback, and curriculum mapping E*Value—clerkship rotation assessments MyProgress—observational assessments of student clerkship performance Medical Education Information System—includes all relevant undergraduate medical education student data such as scholastic standing, wellness participation and surveys, honors and awards, demographics, and biographics PeopleSoft—medical student financial aid data, demographics, and course and grade data
Graduate medical education	ACGME^a^ milestone scores and subcompetency scores Scholarly work (eg, publications and conference presentations) Demographic and biographic data Residency information
Practice data	American Medical Association Physician Masterfile and National Provider Identifier

^a^ACGME: Accreditation Council for Graduate Medical Education.

Within the administrative metadata section of our MEOC website, we created an *Acceptable Use* section to capture a summary of the ethical and appropriate use for each data source. We also identified data owners and data stewards for each data source. The data owners are the individuals ultimately responsible for the ethical and appropriate use of the data included in each source, and the data stewards are the individuals most familiar with the given data source and the context surrounding the data. For example, for admissions-related data sources, the associate dean for admissions is the data owner, and the admissions business analyst is the data steward. Final decisions regarding best practices and acceptable use were decided by the MEOC leadership team after consultation with the data owners and data stewards. Finally, as new data sources were formally vetted and integrated into the MEOC framework, we updated the internal and public data inventory documentation and communicated with the MEOC requestors, data owners and stewards, and other stakeholders.

### Data Request Framework

A comprehensive data request governance framework was created to standardize the intake and processing of requests. The framework was designed to provide a single point of entry for requesters; to provide a method of documenting requests and fulfillment efforts; and to ensure compliance with data privacy, ethical conduct, and human subjects research protections. Every requestor completes a request form, including sign off to a data use agreement that outlines their responsibilities regarding protection of the data. Before any data are released to the investigators for research purposes, documented institutional review board (IRB) approval or exemption is required. When developing our request framework, we identified factors related to requestor expectations: ease of understanding, simplicity of the request Web form and processes, consistency in experience from request to request, reasonable turn-around time, transparency, and ability to see previous requests.

Although advances in technology have led to exponential growth in the ability of medical schools to collect and mine student data, this growth has also led to valid concerns regarding student data privacy [1,9]. One important goal was to apply the current best practices and standardized data protection measures for all our learner data. Example practices followed by the MEOC include providing all data as deidentified, except under specific circumstances; requiring requesters to complete trainings for Family Educational Rights and Privacy Act, Health Insurance Portability and Accountability Act, and information security awareness; requiring evidence of IRB approval or exemption for any data requests related to research; and ensuring that the relevant data owner(s) have the opportunity to approve or deny each request. For research-related requests, the IRB determines requirements for the learner’s consent based on the specific project.

### Data Delivery

MEOC’s default data analysis and delivery method is via Tableau Server [10], a data analytics, reporting, and visualization tool. In addition to being recognized as an industry leader in the space of data analytics platforms, Tableau Server allows technical and nontechnical users alike to easily explore data by using click-and-drag features and filter options. Furthermore, users can easily export data from Tableau Server for import into other tools such as statistical software programs.

### Communication About Medical Education Outcomes Center

A *slow rollout* strategy to communicate about MEOC was implemented. This began with the key internal stakeholders and was then extended to the broader medical school community, including our faculty. We established a dedicated website and intake process as described. A major component of this communication work was done through informational meetings with multiple departments, educational committees, and other key stakeholders. We have also conducted dedicated educational and research-in-progress sessions open to all faculty and staff to discuss the MEOC and to present the research performed using the MEOC resources.

### Ethical Approval

Ethical approval for dissemination of the MEOC model has been granted by the IRB of the University of Minnesota, study number: STUDY00005865. Each research request requires and has received an individual IRB application and approval.

## Results

### Overview

Since fall 2017, the MEOC has fulfilled over 160 data requests, with another 40-plus requests currently in the pipeline. These requests have culminated in a wide range of outcomes analyses, including peer-reviewed publications and support of accreditation and quality improvement work. Building MEOC was an enabling step to accomplish the goals outlined in the Introduction section. Much of this work is currently in progress.

### Examples of Medical Education Outcomes Center’s Projects

#### Predicting Student Outcomes

Data analysis through the MEOC has fostered several projects examining the predictors of medical students’ performance. These projects have used demographic, prematriculation, and exam data to predict performance in medical school as assessed by the grades in foundational science courses, performance on United States Medical Licensing Examination 1 and 2 [11,12], selection to Alpha Omega Alpha Medical Honor Society [13], and the type and location of residency [14].

#### Liaison Committee on Medical Education Accreditation

The MEOC has been used as the data clearinghouse for information needed for the data collection instrument (DCI) as part of the Liaison Committee on Medical Education’s (LCME) reaccreditation work. Requests for DCI data utilized the MEOC’s data request framework and were tracked and completed as described above. These requests have provided a mechanism to complete the DCI’s data tables and to track our current reaccreditation efforts. By standardizing this process, we will be able to prospectively update DCI-related data between LCME accreditation visits and more effectively monitor and report progress addressing any citations. The MEOC has also been used for ongoing continuous quality improvement work for the University of Minnesota Medical School (UMMS).

#### Tracking Graduates

Work has begun on integrating clinical outcomes data into the MEOC to link the effectiveness of medical education programs to future clinical outcomes of UMMS graduates once they enter practice [2-8]. As an initial step in this process, we needed a method to track the UMMS graduates to determine their geographic location and specialty. We used the American Medical Association (AMA) Physician Masterfile, which contains recent practice and training information, including current practice locations, training milestone dates, and certifications. We purchased a subset of the Masterfile, containing 13,092 UMMS graduates, from Medical Marketing Service (Schaumburg, IL), which has been licensed by the AMA to distribute these data.

**Figure 1:**
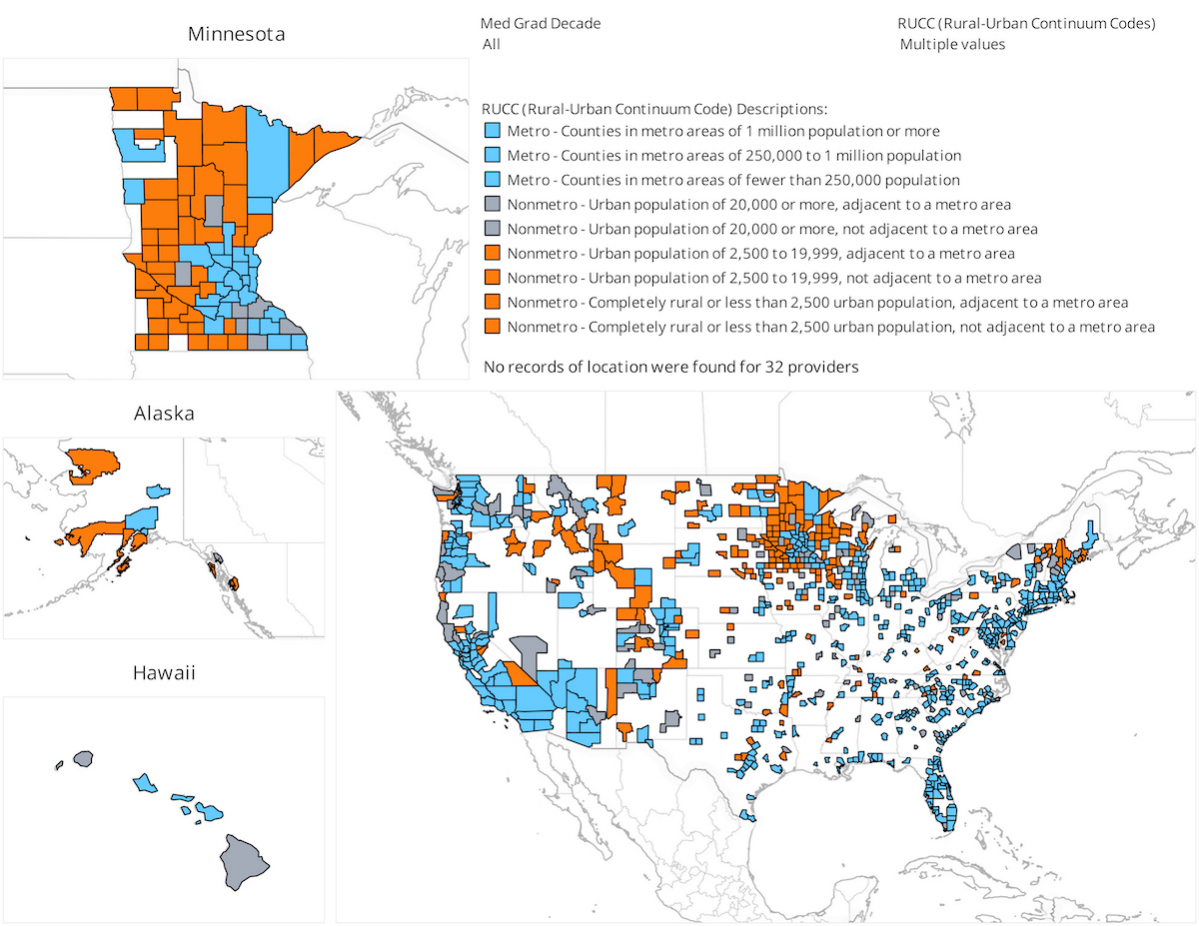
Practice location by county of the University of Minnesota Medical School graduates listed in the American Medical Association Physician Masterfile and that have National Provider Identifier numbers linked to them (n=10,443). Each shaded area represents a single county and may be the location for multiple providers. This figure is created using Tableau software with map data from OpenStreetMap contributors. OpenStreetMap data are licensed under the Open Data Commons Open Database License.

Of the 13,092 UMMS graduates in the Masterfile, National Provider Identifier (NPI) numbers were available for 10,443 individuals. The NPI numbers are issued by the Center for Medicare and Medicaid Services and are used by Medicare and commercial insurers to identify the specific provider of health care services. NPI numbers provide the *key* to link with clinical databases. The geographic distribution of the practice locations for these UMMS graduates with an NPI number is displayed in Figure 1.

Detailed digitized records of our students are kept in the UMMS’s internal Medical Education Information System, with records dating back to 2002. Using data from the NPI registry and a matching algorithm, the MEOC has thus far been able to link 3983 of these student records to the AMA/NPI dataset. With this connection between UMMS-held educational records and AMA/NPI data established, we will be able to link educational measures to clinical databases through NPI numbers to study the effects of medical education on future clinical outcomes down to the level of individual physician data.

## Discussion

### Principal Findings

We developed the MEOC to integrate education data and to build a framework to standardize the intake and processing of requests for using these data. The MEOC has several strengths, as summarized in Table 2. Through the MEOC, requests for data are generated, documented, and tracked in a formal, streamlined, and consistent manner. Prior requests for similar data or for similar purposes are leveraged, leading to greater efficiency. Formalization of these processes mitigates security concerns surrounding data delivery, privacy, and access.

**Table 2:** Strengths of the Medical Education Outcomes Center (MEOC). This table outlines the common problems faced before and after the development and implementation of the MEOC framework.

Problem	MEOC’s solution
Uncertainty about where and how to request and obtain data	Single point of entry for all data requests
Inconsistent, informal, or undocumented processes for requesting and providing data	Formal, documented, streamlined, and consistent processes to generate and track all data requests, including associated approvals, rationale, and permissions tracking
Uncertainty regarding what data are needed or are available and relevant for a requestor’s specific needs	Knowledge and guidance in identifying proper data sources and data elements
Use of the same data for similar purposes, resulting in potential duplication of effort and inefficiencies	Prior requests for similar data or purposes are leveraged, leading to greater efficiency, consistency, and potential opportunity for collaboration
Independent or solo analysis and interpretation of data, potentially with limited context or experience	Full range of services to assist in analyzing and interpreting data
Errors or inconsistencies in the definition, use, and interpretation of data	Development of standardized data definitions, fostering the consistency in use, definition, and interpretation of data throughout the school
Data residing in siloed databases	A framework for the integration of disparate data sources
Potential for privacy and security concerns surrounding data delivery and access	Secure data delivery methods with ethical, data privacy, and human subjects research protections compliance, including proper deidentification protocols
Difficulty tracking learners as they progress along the medical education continuum into practice	Use of the American Medical Association Physicians Masterfile and National Provider Identifier numbers to link learner data and educational measures to clinical outcomes

### Challenges and Limitations

An initial challenge in building the MEOC was related to stakeholder engagement. For example, we needed to formalize the roles of data owners and data stewards, demonstrate the value of this project to them, develop an effective communication strategy, and streamline the work they needed to do. An important component of this initial work was to define the governance structure for the use of the data. Initial technical challenges included identifying and integrating the many sources of data that are owned or utilized by the medical school; optimizing our website and data request framework; and conducting back end data work including establishing or optimizing databases and performing data quality assurance, normalization, and cleaning efforts.

Procurement of resources for supporting the work in the MEOC was another challenge. Since its inception, the MEOC has been supported by operational funds, as discussed above. As the MEOC became more established, we experienced capacity issues. As stakeholders throughout the medical school became aware of the MEOC, the number of data requests has increased, and we have required more personnel to be able to meet these increased demands. Furthermore, although the MEOC data framework has worked very well to date to provide flexibility in the data model, the process of combining data sources for every request is resource heavy and limits the scalability of the center. Therefore, we are now exploring the possibility of also building a data warehouse, which would include the elements most frequently needed by our users.

### Next Steps

The major next step is to link educational measures and outcomes data to clinical databases using the NPI numbers of our students and residents. This will allow us to develop predictive models for future career choice, practice location, and, ultimately, clinical performance of our graduates. We will be able to begin assessing the effectiveness of our medical education programs by the quality of care delivered by our graduates [2,4,5,7,8]. Linking the medical education continuum to clinical practice will be a powerful tool to facilitate the design of educational experiences that positively impact patient outcomes, a link of utmost importance that has yet to be broadly formed. We will be able to determine if educational experiences, such as rural longitudinal integrated clerkships, are a more effective training model with lasting effects (ie, *educational imprinting*) and whether this model impacts rural versus urban practice location. We will be able to determine whether it matters where one trains and how long the training effects persist. We will be able to study the impact of specific curricular elements on future practice patterns and apply predictive analytics to prematriculation data to select students who meet the goals of our school. Many additional questions will now be accessible for study, and the MEOC will also aid in the design of future studies to ensure these questions and the studies to answer them are well designed.

Several publicly available clinical practice data are available on a local and national level such as Medicare data, clinical registries, and health system databases [2,7]. Linking educational and clinical data raises important challenges such as the attribution of outcomes in complex and interprofessional health systems, the lag time between education and practice, the tracking of learners across institutions, and the long-term impact and sensitivity of educational interventions on clinical outcomes [2,3,5,7,8].

Despite the challenges of linking education data to practice data, some associations between medical education and clinical outcomes have been reported, illustrating the power and potential of this type of work. Asch et al have evaluated obstetrical residency programs using maternal complication rates and demonstrated that residents trained in programs with low maternal complication rates had lower complication rates in subsequent practice [4,15]. Chen demonstrated a relationship between the spending patterns in the region of a resident’s Graduate Medical Education program and the subsequent mean expenditure per Medicare beneficiary by that resident, once they entered the internal medicine or family medicine practice [16]. Associations have also been demonstrated among the licensing exam scores, delivery of lower-intensity clinical care, quality of surgical residency programs, and the future practice performance of graduates [17-19]. To more effectively link medical education to clinical practice, a uniform system for collecting and analyzing outcomes and greater availability of prospectively designed databases that can be used across institutions are needed.

### Conclusions

In summary, the MEOC provides a model for the development of an educational outcomes center that can be adapted to other institutions. The MEOC’s integration of data sources and data request framework provides greater accessibility to data to inform medical education practice and research. By using the MEOC framework as a model, medical schools can leverage their data and related analytic resources to more effectively operate their programs and drive innovation.

## Abbreviations

AMA: American Medical Association
DCI: data collection instrument
IRB: institutional review board
LCME: Liaison Committee on Medical Education
MEOC: Medical Education Outcomes Center
NPI: National Provider Identifier
UMMS: University of Minnesota Medical School
